# Biodegradation of Some Organic Materials in Soils and Soil Constructions: Experiments, Modeling and Prevention

**DOI:** 10.3390/ma11101889

**Published:** 2018-10-02

**Authors:** Andrey V. Smagin, Nadezhda B. Sadovnikova, Vyacheslav I. Vasenev, Marina V. Smagina

**Affiliations:** 1 Soil Science Department, Lomonosov Moscow State University, GSP-1, Leninskie Gory, Moscow 119991, Russia; nsadovnik@rambler.ru; 2 Institute of Forest Science, Russian Academy of Sciences (ILAN), 21, Sovetskaya, Uspenskoe, Moscow 143030, Russia; vasenyov@mail.ru (V.I.V.); msmag08@mail.ru (M.V.S.); 3 Department of Landscape Design and Sustainable Ecosystems, Agrarian-technological Institute, Peoples’ Friendship University of Russia, Miklukho-Maklaya St., 6, Moscow 117198, Russia

**Keywords:** biodestruction, soil constructions, sustainability, polymers, synthetic hydrogels, peat, CO_2_ emission, water-retention, modeling

## Abstract

The decomposition of natural and synthetic polymeric materials (peat, humates, biochar, strongly swelling hydrogels and other soil conditioners) in a biologically and chemically active soil environment inevitably leads to a reduced ability to improve the structure, water-retention, absorptive capacity and fertility of artificial soil constructions in urbanized ecosystems and agro landscapes (constructozems). Quantitative assessment of the biodegradation process using field and laboratory incubation experiments, as well as mathematical modeling, showed the possibility of significant (up to 30–50% per year) losses of organic matter of constructozems and a corresponding deterioration of soil quality. Incubation experiments that track the carbon dioxide emission rates of polymeric materials under given thermodynamic conditions allow for the estimation of decomposition rates in addition to an exploration on the dependence of such rates on additions of microbial inhibitors. The use of nomographs provide an opportunity to optimize long-term amendment performance in soil constructions by identifying the most favorable depths to apply amendments to ensure stable functioning during desired in-service timelines in the built environment. The results of the study are useful for geo-engineers and landscaping practitioners.

## 1. Introduction

The synthesis and decomposition of organic matter significantly influences the formation and functioning of soils. Anthropogenic perturbations to these processes arising in agro-ecosystems, urban, and military landscapes (SUITMA’s), commonly leads to soil degradation, deterioration of physical, chemical and biological properties, loss of fertility and other environmental functions [1,2,3]. The lack of organic substances in such soils must necessarily be compensated by applying soil conditioners that may be of either organic or synthetic manufacture. It is important to evaluate not only the effect of organic materials on soil properties, but also their stability or characteristic residence time in a biologically and chemically active soil environment [4,5]. 

In the first (linear) approximation, the following model for the destruction of organic matter with a concentration (inventory) (*C*, kg/m^2^) and a flux (input) on the surface (*L*, kg·m^−2^·year^−1^) can be used for this purpose [6,7].

(1) $$\frac{dC}{dt}=L-kC,$$

where *t* is the time, *k*, year^−1^ is the mineralization (biodegradation) constant associated with the half-life of the substance:

*T*_0.5_ = ln2/*k*, (2)

and with a 95% decay period:

*T*_0.95_ ≈ 3/*k*. (3)

The model (1) and expressions like (2) and (3), which follow from it, underlie the estimation of the characteristic times of soil organic matter turnover. Raich and Schlesinger [8] estimated a turnover time for organic carbon in soils that ranged from 10 years in tropical grasslands to 500 years for tundra and wetland environments, with a global average of 32 years. According to [9], the average turnover time of 75% of the organic matter of tropical forest soils does not exceed 30 years with a total carbon stock of 14–27% of the global soil reservoir. In the review [10], the average time of carbon turnover in the soil cover of the planet is estimated at only 20–22 years. The radiocarbon dating of humus from the upper 10 cm of the thickness of the zonal series of Eurasian soils from the red soil of moist subtropics (Cambisols Chromic) to the arctic tundra soil (Cryosol Haplic) gives characteristic times of organic matter formation in 60–350 years [11]. In experiments on the peat mineralization of various bog ecosystems from the tropics to northern latitudes, the characteristic time of 95% organic matter decomposition (*T*_95_) was 50–250 years, and the half-life was 5 < *T*_50_ < 50 years [12,13]. The value of the litter-and-fall coefficient (*L*/*C*), which estimates the turn-up time of detrital carbon in the first approximation, according to model (1) varies from 0.2 to 25 years, and the *T*_95_ index from 1.3 to 60 years [6,14,15]. Thus, the dominant part of the organic matter of the upper (fertile) soil layers is deposited there for a rather short period. Only removed from the surface or buried organic substrates can persist for centuries and even millennia, as shown by the radiocarbon dating of individual fractions of soil humus [3,11]. At the surface of the soil in the zone of the maximum biological and biochemical activity, the decomposition of carbon stocks including humic substances, peat, lignin, and biochar, takes place at a relatively rapid pace, which is probably promoted by the priming effect [16,17,18,19].

These natural patterns of dynamics and stability of organic substances in soils should be taken into account in calculations of doses of organic fertilizers and soil conditioners, as well as in the design of soil constructions in anthropogenic ecosystems. First of all, we must understand that if there are absolutely no stable organic substances, artificial soils (constructozems) cannot be expected to perform their intended tasks in-perpetuity. However, two strategies are commonly used to prolong the life span of the fertile layer of soils with organic conditioners. The first way is to reduce the susceptibility of soil conditioners to biodegradation by including in their composition substances that inhibit biological activity. Such a method, from our point of view, is particularly effective for synthetic polymer hydrogels, which can simultaneously be used to control pathogenic microflora in urban and agricultural soils. The suppression of pathogenic microorganisms in the rhizosphere is possible through the creation of local gel structures with modern plant protection products, for example, based on silver ions or nanoparticles [20]. Unlike aqueous solutions and suspensions, gel structures are firmly fixed in the root zone, thus preventing vertical migration of water and removal of water-soluble substances, including plant protection agents [4,21]. For toxic agents, this means a decrease of the ecological risk of contamination of the adjacent environments (i.e., soil and groundwater). Given resistance to degradation and seasonal persistence in the root zone, hydrogels retain their ability to improve the water-retaining and absorbing capacity of soils. Their use in soil engineering allows extending a period of steady water consumption (without reduction of transpiration) of plants in drought conditions for 2–3 weeks or to achieve 1.5–2-fold water savings during crop irrigation [4,21]. 

The second way of preserving organic substances consists in their removal from the surface zone with the maximum biological and biochemical activity [4,21]. Microbiological activity usually decreases exponentially with the depth of the soil, which is apparently due to a similar pattern of distribution of root systems, carbon substrates and oxygen, limiting the functioning of aerobic microflora [2,22]. This circumstance theoretically gives the opportunity to determine the optimum depth of localization of organic conditioners in the soil, which guarantees their stability for a given service life [4]. 

The main objective of the study was to quantify and simulate the biodegradation of the most effective soil conditioners for urban landscaping and irrigated agriculture in the form of natural (peat) and synthetic (polymer hydrogels) organic materials. Specific tasks included:

- Determination of the rate of destruction of peat in laboratory incubation experiments, depending on the control factors of temperature and humidity, and mathematical modeling of their effects;
- Quantitative evaluation of biodegradation of different types of strongly swelling polymer hydrogels in laboratory incubation experiments under the influence of inhibitors of biological activity based on ions and silver nanoparticles;
- Quantitative assessment of the effect of soil conditioners and their biodegradation on the physical state and water-retaining capacity of soils;
- Determination of the intensity of destruction of biopolymer materials in the field;
- Determination of the dependence of biological activity of soils on depth, justification and verification of the nomographs method for estimating the reduction of biodegradation of soil conditioners at a given depth of soil.

## 2. Materials and Methods

Natural soils (Albeluvisols Umbric, Arenosols) and special soil constructions (urban constructozems) were investigated in humid climatic conditions on the territory of the Moscow megapolis (western administrative district), at the Experimental Station of the Moscow State University “Chashnikovo” and in the arid zone at the Experimental Station of Qatar Ministry of Municipal and Agricultural Affairs near Doha. Soil samples were taken from different depths within 1 m of soil by special soil auger for simultaneous determination of water content (*W*_%_) and soil bulk density (ρ*_b_*, g∙cm^−3^):

(4) $$W_{\%}=100\frac{m_{w}-m_{s}}{m_{s}}$$

(5) $$\rho_{b}=\frac{m_{s}}{V_{t}}$$

where *m_w_*, *m_s_* and g are the masses of the sample before and after drying at 105 °C, *V_t_*, cm^3^ is the volume of soil auger. In addition to soil samples in some experiments, calcined quartz sand was used as a model soil substrate devoid of organic matter. 

As soil conditioners, peat samples from various deposits (Tver, Moscow, Tomsk regions of Russia) before and after mechanical dispersion, as well as strongly swelling polymer hydrogels (SSPH) were tested. The hydrogel compositions were prepared on the basis of 5 types of strongly swelling polymers: *№*1—acryl hydrophilic Aquasorb preparation (France) with high degree of swelling (HDS) in pure water (700–1000 gH_2_O/g); *№*2—radiation-cross-linked technical polyacrylamide, synthesized in Institute of Chemical Physics of the Russian Academy of Science, with HDS in pure water 700–1000 g/g, and in saline solutions of 0.01–0.1*n* concentration at least 250–500 g/g; an experimental batch of acrylic gels with HDS in pure water 500–700 g/g from the Ural Chemical Company, prepared using proprietary technology [23] that included samples *№*3—with hydrophilic filler in the form of waste biocatalytic production of polyacrylamide, *№*4—with amphiphilic excipient in the form of humates, and the addition of microelements, *№*5—with amphiphilic excipient in the form of dispersed peat. 

Silver, as an inhibitor of biological activity, and as a component of protective gel compositions in the form of nanoparticles (experienced “Zeroxxe” of AgroChimProm GC (http://tdahp.ru/en/) with amphiphilic surfactant-stabilizers [24] and in the form of ionic (chemically pure AgNO_3_) solution were added to SSPH in the concentration (for the active substance, Ag) 10, 100, 1000 ppm per mass of water in swollen hydrogel. Variants of the experiments with silver inhibitors included pure gel compositions, and their mixtures with water extracts of rotting potato tubers and calcined to 500 °C sandy substrate.

The main method of laboratory testing was incubation of samples in closed vials with subsequent assessment of soil respiration by CO_2_ emission. The index of specific or so-called basal respiration, obtained in incubation experiments, is often used to assess the activity of the microbial community of soils, including in urban settings [2,25,26]. However, despite the standardization of the definition of this indicator [27], a number of researchers indicate serious methodological problems not solved in this standard [2,25]. Therefore, in the following we describe the modified method for determining soil respiration according [2], which was used in our study. In closed vials of dark glass or plastic with soil samples, proper conditions were created for the development of microorganisms and biodegradation of organic substrates. For this purpose, the previously calculated amount of water was added to air-dry soil samples and their compositions with soil conditioners to obtain optimum moisture content 0.7–0.8 units from the total moisture capacity (*W_s_*). This value was estimated by calculation from the data on soil bulk density (5) and the density of the solid phase (ρ*_s_*, g/cm^3^) by the formula:

(6) $$W_{s}=\left(\frac{\rho_{w}}{\rho_{b}}-\frac{\rho_{w}}{\rho_{s}}\right)$$

where ρ*_w_*= 1.0 g∙cm^−3^ is the density of water, ρ*_s_* = 1.6 g∙cm^−3^ for peat and ρ*_s_* = 2.65 g∙cm^−3^ for mineral soil substrates. In experiments with gel-silver compositions the humidity was determined according the degree of swelling of SSPH 100 g/g in pure gel compositions that in soil-gel mixes gives 1:1 ratio of water and mineral components.

Moist samples in tight-sealed flasks were places in a thermostat with the optimum temperature of incubation of 25–30 °C. The optimum humidification and temperature conditions corresponded to the highest or potential biological activity and biodegradation of organic substrates in a given soil sample. In separate experiments, biological activity (respiration) and biodegradation were investigated at different humidity levels (0 < *W* ≤ *W_s_*) and temperature (4 ≤ *T* ≤ 30 °C) simultaneously with the thermodynamic analysis of the water-retaining capacity of the soil by centrifugation [4,28] (see below). After the time interval of incubation (Δ*t* = 20–26 h), changes in the contents of CO_2_ (Δ*X_%_*) compared to the initial (atmospheric) level were measured, and the soil respiration rate (*U_m_*, mgCO_2_∙kg^−1^∙h^−1^) was calculated per unit mass of the soil-substrate solid phase (*m_s_*) using the following equation [2,4]:

(7) $$U_{m}=\frac{PMV_{g}\Delta X_{\%}}{100RTm_{s}\Delta t}$$

where *R* is the universal gas constant (8.314 J∙mole^−1^∙K^−1^), *T*—absolute temperature, K, *P*—atmospheric pressure (Pa), *M* = 44 g∙mole^−1^—molar mass of CO_2_, *V_g_*, mL—volume of the gas phase in the flask.

The volumetric content of CO_2_ in air samples from the flask was determined using a PGA-7 infrared gas analyzer (Electronstandart, Saint-Petersburg, Russia). The Δ*X*_%_ value for CO_2_ was estimated by the modified method [2,20] involving correction for the adsorption and dissolution of gas generated by microorganisms during incubation. In this method, after the end of incubation, flasks were placed in a microwave oven ME83XR (Samsung Electronics Port Klang, Malaysia) for 3 min for degassing of soil solution and thermo-desorption of CO_2_ from the surface of soil particles by heating to 70–80 °C. 

The content of organic carbon (*C*_%_) in the samples was determined by coulometric titration using the AN-7529 analyzer (Gomel Plant of Measuring Devices Gomel, Russia). Calculation of biodestruction constant (*k*, year^−1^) from the data of the CO_2_ emission (*U_m_*, mgCO_2_∙kg^−1^∙h^−1^) and percentage carbon content (*C*%) in soil conditioners was carried out according to the linear model of organic matter biodegradation (1) in the form of the following ratio [20]:

(8) $$k=\frac{T_{b}}{T_{0}}\left(\ln100-\ln\left(100-24\cdot 10^{-2}\frac{12U_{m}}{44C_{\%}}\right)\right)$$

where *T*_0_ = 1 is the time scale for the process of biodegradation in years, 12/44 is the ratio of the molar masses of carbon and CO_2_, 24 × 10^−2^ is the conversion factor from hours to days, from milligrams to kilograms and from % to kg/kg, *T_b_* is the average yearly period of biological activity, expressed in days.

Thermodynamic analysis of water-retention capacity of soil samples and their compositions with soil conditioners was carried out by centrifugation method, with modifications [4,20], using a laboratory centrifuge CLS-3 in the range of water-retention energy (soil water potential or equivalent pressure) from 0 to 800–1000 J/kg (kPa). As a basic criterion, the water retention curve (WRC) or the dependence of the thermodynamic potential of water from its content in the soil is used. Approximation of experimental data of WRC has been performed by van-Genuhten [29] model. 

Field experiments on the destruction of soil conditioners were conducted at Moscow in layered soil constructions based on peat soil-modifiers and hydrogels [4,21]. The decomposition of peat was determined in capsules from nylon fiber by decreasing the mass of peat samples dried at 95 °C for a certain period of time (*t*). The mass ratio before (*m*_0_) and after the fixed stage of the experiment (*m_t_*), characterized the biodegradation rate:

*D*_%_ = 100*m_t_*/*m*_0_. (9)

After completion of all successive stages of the experiment, the dependence of *D*_%_ on time was obtained. An estimate of the biodegradation constant (*k*) averaged over the entire period or the characteristic times (*T*_0.5_ and *T*_95_) of the biodegradation process, in accordance with (2) and (3), was obtained using the model (1). The destruction of SSPH was analyzed in the same way, only not by weight loss, but by reducing the content of organic carbon in capsules with calcined quartz sand mixed with hydrogel. Hydrothermic parameters were controlled in an automatic mode by programmable sensors “Hygrochron” DS1923 (Dallas Semiconductor, Dallas, TX, USA) and loggers Decagon (USA).

All experiments were carried out in 3–4 multiple repetitions with subsequent statistical and mathematical processing of data using standard functions of MS Excel and the nonlinear regression wizard of S-Plot 2001 software for mathematical modeling. Numerical modeling of biodegradation was carried out in the Matlab-7 software.

## 3. Results

### 3.1. The Study and Modeling of Peat Biodegradation

**Figure 1:**
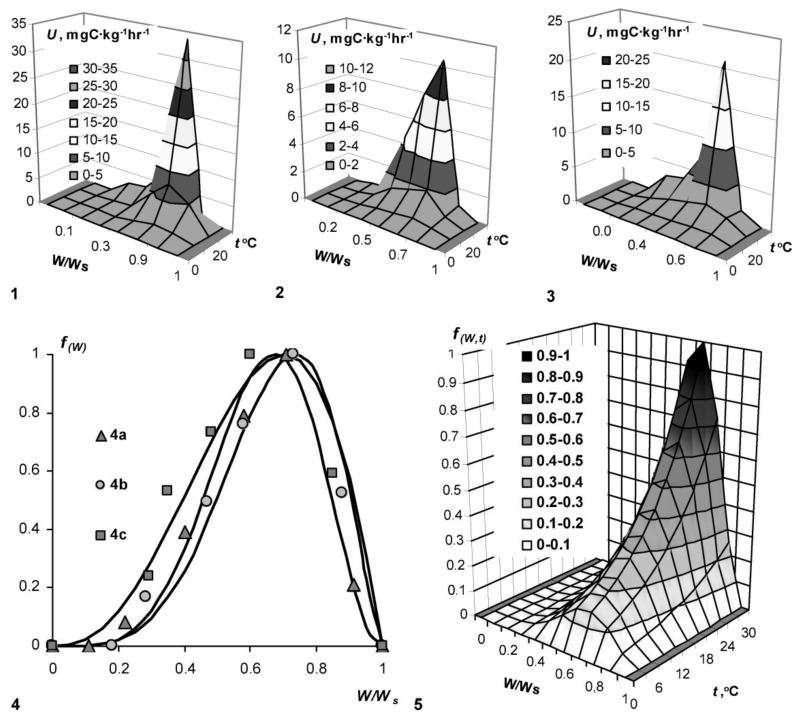
Quantification and modeling of decomposition rate of organogenic substrates (U) as a function of temperature (*t*) and relative humidity (*W*/*W_s_*). 1—peat (10 cm), Tver region, 2—peat (100 cm), Tomsk region, 3—peat (10 cm), Moscow region; 4—model (11): 4a—peat (100 cm), 30 °C, Tver region, *f*_(*W*)_ *=* (*W*/*W_s_*/0.67)^4.84^{(1 − *W*/*W_s_*)/(1 − 0.67)}^2.35^; R^2^ = 0.96, s = 0.07; 4b—peat (100 cm), 5 °C, Tomsk region, *f*_(*W*)_ *=* (*W*/*W_s_*/0.74)^4.27^{(1 − *W*/*W_s_*)/(1 − 0.74)}^1.53^; R^2^ = 0.99, s = 0.03 4c—peat (10 cm), 20 °C, Moscow region, *f*_(*W*)_ *=* (*W*/*W_s_*/0.70)^2.54^{(1 − *W*/*W_s_*)/(1 − 0.70)}^1.09^; R^2^ = 0.99, s = 0.04; 5—model (12), peat (100 cm), Tver region.

**Figure 2:**
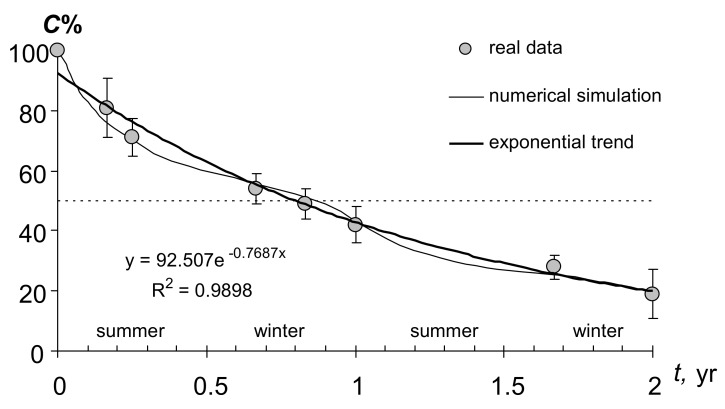
Biodegradation of peat (2-year experiment) on the surface of urban soil in the Moscow megacity.

**Figure 3:**
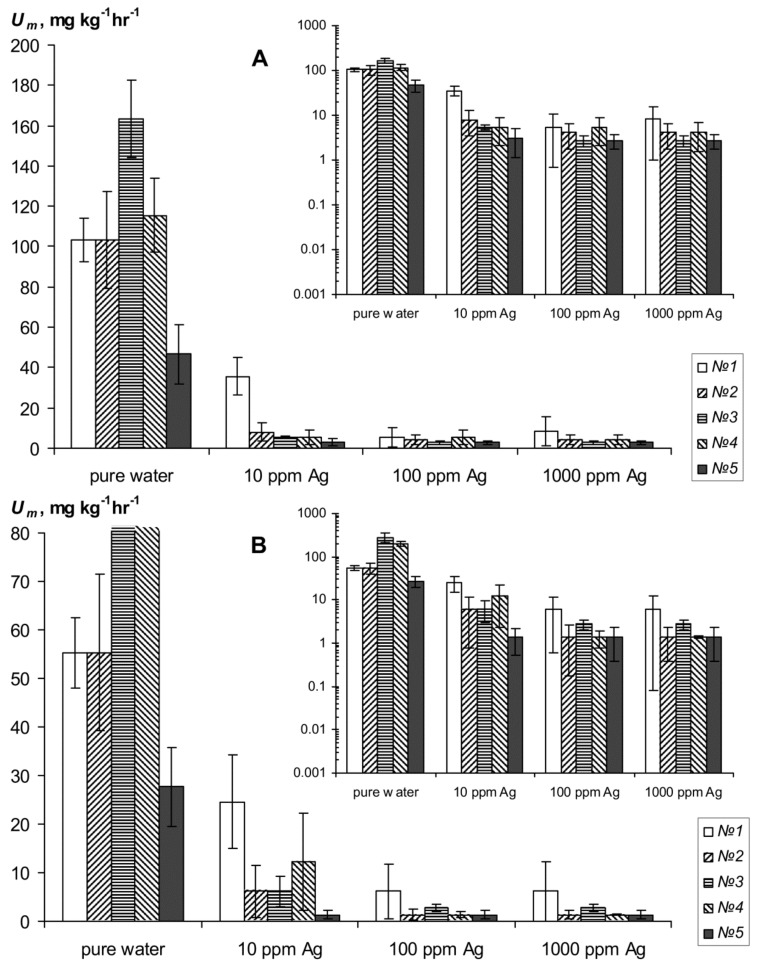
Carbon dioxid emission as an indicator of resistance to biodegradation of the hydrogels and their compositions with silver. *№№*1–4 are different types of hydrogels (see “Objects and methods”); (**A**) is ionic silver and (**B**) colloidal silver; insets are semi-logarithmic scale.

**Figure 4:**
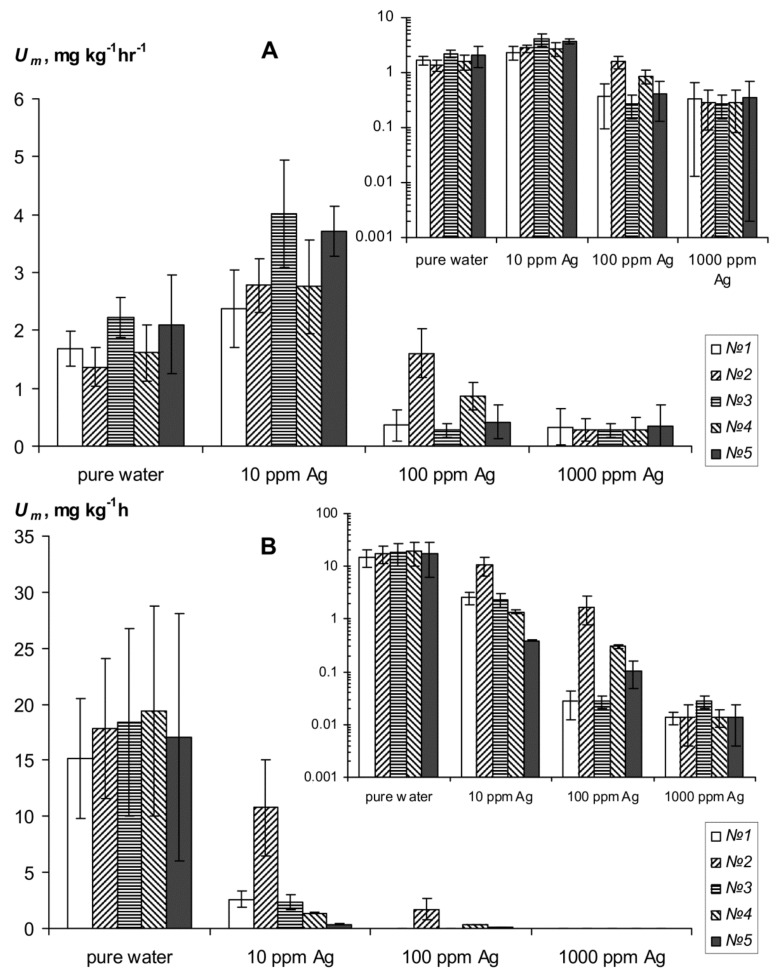
The change in *U_m_* of hydrogels and gel compositions with silver in the case of mixing of root rot and sandy substrate; *№№*1–4 are different types of hydrogels (see “Objects and methods”); (**A**) is ionic silver and (**B**) colloidal silver; insets are semi-logarithmic scale.

A quantitative assessment of the intensity of biodegradation (microbial respiration) in peat samples of different genesis reveals a nonlinear form of the dependence of these processes on the controlling factors—temperature (*T*, °C) and humidity (dimensionless index *W*/*W_s_*, where *W_s_* is the total moisture capacity). (Figure 1). The dependence is represented by a function with an extremum in the range 0.6–0.7 units of *W*/*W_s_* and 25–30 °C of temperature. To describe the dependences obtained in the temperature range from zero to 30 °C and relative humidity (0 < *W*/*W_s_* ≤ 1), the following formulas are proposed [4]:

(10) $$U_{\left(T\right)}=m_{\left(T\right)}U_{max};\;m_{\left(T\right)}=Q_{10}^{\frac{T-T_{m}}{10}}$$

where *U_max_* is the maximum destruction intensity at the optimum, *T_m_* is the optimum temperature (30 °C), *Q*_10_ is the temperature coefficient, which on the average can be taken equal to two;

(11) $$U_{\left(W\right)}=f_{\left(W\right)}U_{max};\;f_{\left(W\right)}={\left(\frac{W}{W_{m}}\right)}^{a}{\left(\frac{1-W}{1-W_{m}}\right)}^{b}$$

where *W_m_* = *a*/(*a* + *b*) is the water point of the extremum (*U_max_*) on the curve of the *U*_(*W*)_ dependence, *a*, *b* are empirical constants. Estimation of empirical parameters *a* and *b* from experimental data is carried out using the nonlinear regression software package in the S-Plot computer program (see examples in Figure 1, Figure 2, Figure 3 and Figure 4). High coefficients of determination (R^2^ = 0.96–0.99) at small standard approximation errors (*s* = 0.03–0.07) and statistically significant at level *p* < 0.001 the parameters *a*, *b* confirm the adequacy of the model. The relationship between the parameters *a* and *b* with the extremum point *W_m_* simplifies the estimation of these values and makes the model actually two-parameters. 

Simulation of the total contribution of temperature and humidity to the process of destruction of organic substances and the release of gaseous carbon can be achieved using a combination of functions (10) and (11). In the simplest case, it is sufficient to multiply the factors of humidity *f*_(*W*)_, temperature *m*_(*T*)_ and the maximum value of the biodegradation rate (*U_max_*) under optimum conditions (*T = T_m_*, *W = W_m_*), i.e.,

U = f_(W)_·m_(T)_·U_max_. (12)

**Figure 5:**
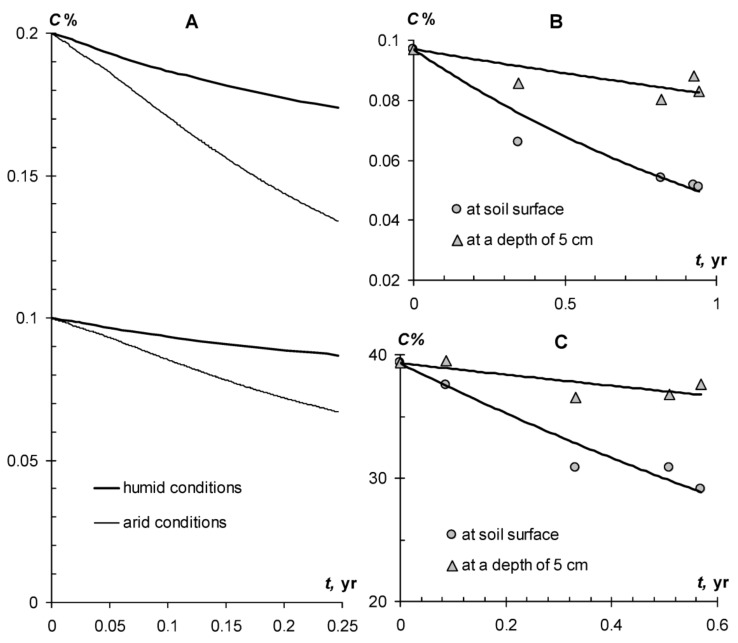
Modeling and experimental evaluation of biodegradation of organic soil-modifiers under different conditions. (**A**) modeling of biodegradation of SSHP (doses 0.1 and 0.2%) depending on temperature in humid and arid climates. (**B**) biodegradation of hydrogel, (**C**) biodegradation of peat soil modifier in experimental soil constructions for urban lawns (Moscow). The equations of exponential regression as an analytical solution of (1): (**B**) (at soil surface): *C*% = 0.1·exp(−0.712·*t*), R^2^ = 0.93; (**B**) (at depth of 5 cm): *C*% = 0.1·exp(−0.171·*t*), R^2^ = 0.69; (**C**) (at soil surface): *C*% = 39.3·exp(−0.543·*t*), R^2^ = 0.93. (**C**) (at depth of 5 cm): *C*% = 39.3·exp(−0.118·*t*), R^2^ = 0.64.

An example of such a calculation is shown in Figure 1, Figure 2, Figure 3, Figure 4 and Figure 5.

Analysis of the obtained quantitative patterns of biodegradation (Figure 1) shows that a relatively small decrease in humidity (up to 0.6–0.7 units from the total moisture capacity) leads to a sharp burst of biological activity and the destruction of organic matter of peat. This may be the reason for the disappearance of peatlands [1,16] during drainage melioration, as well as rapid biodegradation of urban green lawns on peat and peat-mineral soil substrates during their intensive irrigation. Under hydrothermic optimum conditions, organogenic materials can be removed up to 20–30 mgC/kg·h (Figure 1). With a typical content of organic carbon at 30–40% of the total detritus mass, calculation according to (8) gives an estimate of the peat biodegradation constants *k* = 0.4–0.9 year^−1^ at *T_b_* = 365 days. That is, at the maximum possible rate of destruction under optimum conditions, the half-life of peat will be only *T*_0.5_ = ln2/*k* = 0.8–1.6 years, and 95% of peat will decompose during the time *T*_0.95_ ≈ 3/*k* = 3–7 years. 

In reality, the optimum conditions cannot exist in the soil for such a long time, and in the calculations it is necessary to use the period of biological activity *T_b_* < 365 days, and also to take into account the dynamics of the controlling factors *T* and *W*. However, direct observations of peat destruction on the soil surface in urban conditions show that the real value of *k* = 0.77 year^−1^ is close to the upper border of the range obtained in the laboratory for the potential destruction *k* = 0.4–0.9 year^−1^ (Figure 2). This value was obtained by approximating the data of a two-year experiment on the decomposition of peat on the surface of constructozem in humid conditions in Moscow using the model (1). In accordance with the exponential model, especially large peat losses were observed in the first (summer) months of the experiment. At this time, destruction can reach 9–13% of organic matter per month. In winter, the process slows down somewhat, but does not stop completely. The loss of peat at this time is 3–4% of organic matter per month, and the total destruction during the cold season reaches 10–15% of organic matter or 20–25% of the total destruction in the first year of the experiment as a whole. A more correct description of peat biodegradation during the year is provided by the numerical model [4,5], implemented in the Matlab-7 program. In this model, the kinetic parameter of destruction (*k*) is not constant and depends on the controlling factors of temperature and humidity in accordance with (10) and (11): *k* = *k_max_*·*f*_(*W*)_·*m*_(*T*)_. Known monitoring data on the annual dynamics of the parameters *T* and *W* in the Moscow megapolis [4,26] are introduced into the model by spline-approximation [5]. The results of the numerical simulation are shown in Figure 2. They clearly reflect the increase in biological activity in the summer with a rise in temperature and a decline in the cold season.

In just first year, the peat substrate loses up to 50–60% of its mass (Figure 2). In the future, the process gradually slows down, in accordance with the model of biodegradation (1). The half-life and characteristic time of 95% organic matter decomposition, estimated by Equations (2) and (3) are 0.9 and 3.9 years respectively. Such a low stability of peat substrates actively used in the practice of gardening in Moscow, inevitably lead to degradation of vegetation, in particular green lawns [26]. Moreover, the destruction of unstable peat substrates leads to an additional release of carbon dioxide into the atmosphere and a general negative carbon balance of urban lawns [26], exacerbating the local greenhouse effect. The specific intensity of CO_2_ emission from a unit of area during annual peat decomposition (200–500 g·m^−2^ year^−1^) exceeds the analogous indicator of anthropogenic release (100 g·m^−2^·year^−1^ from the calculation of 100 thousand tons per year of exhaust gases in the area of Moscow about 100 thousand hectares). In order to compensate for this source, according to model (1), the net productivity (*L*) in the stationary state should be not less than 200 g·m^−2^·year^−1^, and the gross productivity of the lawn is not less than 400 g·m^−2^·year^−1^ of dry mass, taking into account a 50% loss of respiration, which is hardly achievable using conventional lawn grass mixtures. 

### 3.2. The Study and Modeling of the Hydrogel’s Biodegradation 

The experimental results of strongly swelling polymer hydrogelsSSPHs biodegradation under the influence of silver inhibitors of the biological activity are shown in Figure 3 and Figure 4. The magnitude of CO_2_ emission of pure SSPHs ranged from 28 ± 7.2 to 280 ± 72 mg∙kg^−1^∙h^−1^, which meant high and very high biological activity, in accordance with known criteria for [2]. This activity is enough to cause considerable damage to SSPHs organic materials in the soils. The half-time period of microbial decay of SSPHs ranged from 0.5 to 2.6 years and only in the sample *№*5 with amphiphilic fillers in the form of dispersed peat it was 5.2 ± 1.5 years. Mixing of SSPHs with calcined sand and extracts of root rot greatly reduced the emission of CO_2_ to values from 2.0 ± 0.9 to 19.4 ± 11.0 mg∙kg^−1^∙h^−1^ (Figure 4). However, the biodegradation of SSPHs in such compositions, on the contrary, increases, probably due to an increase in the aeration and in the speed of colonization by microorganisms of the hydrogel within the coarsely textured macroporous substrate as compared to a pure hydrogel which is practically impermeable to air and microbes. Calculation by formulas (8) and (2) with the conditional period of biological activity of *T*_b_ = 200 days results in the values of *T*_0.5_ from 0.2 ± 0.1 to 1.1 ± 0.2 years, which is 2–5 times less than in pure hydrogels. The typical time of 95% organic matter decomposition *T*_0.95_ varies for pure SSPHs from 2.2 ± 1.1 to 22.6 ± 6.5 years, and for mixtures with sand from 0.4 ± 0.8 to 4.6 ± 3.0 years.

Low resistance of SSPHs to microbial decay in soils is confirmed by direct observations and forecast on the mathematical models of biodegradation [5]. A separated five-month-long incubation experiment in calcined quartz sand and polymineral sandy arenosol was performed for assessing the biodestruction rate of radiation-cross-linked technical polyacrylamide (sample *№*2) in thermostats at constant temperatures of 20, 30, and 37 °C, which simulated different biodestruction conditions in the humid and arid climatic zones. According to the exponential model of (1–3) the destruction rate obtained in the experiment at 37 °C temperature corresponded to biodegradation constants *k* of 0.7–2.7 year^−1^ or SSPH half-life values of *T*_0.5_ = 0.3–1.0 years. Almost complete destruction (95% of the SSPH) will be attained at this rate within 1.1–4.3 years according to the parameter *T*_0.95_ (3). At this destruction rate, the effect of the SSPH apparently disappears within the first year after its addition in most cases. The incubation of the samples at 30 °C slightly decreased the degradation rate of the SSPH and kinetic constants of biodegradation, reduced here to 0.4–1.7 year^−1^, giving half-lives *T*_0.5_ = 0.4–1.7 years and *T*_0.95_ = 1.8–7.5 years. The highest stability of the SSPH was revealed in an experiment at room temperature (about 20 °C). The destruction rate was characterized by kinetic constants *k* of 0.2–1.5 year^−1^, or the SSPH degradation half-lives *T*_0.5_ = 0.5–3.5 years and *T*_0.95_ = 2–15 years.

The generalized results of this experiments allowed revealing the temperature (*T*) dependence of the kinetic degradation constants of the SSPH [5]. It had an exponential form and was described by the following equation: *k*(*T*) = 0.2834·exp(0.0435·*T*). From the obtained relationship, the potential degradation of the hydrogel was calculated using the exponential model (mentioned above) of biodestruction [5]. Since hydrogels always contain water available for microorganisms, only the temperature dependence of the degradation was taken into account in this simplified version of the model. The temperature was taken as a function of time (*T*(*t*)) from the actual data obtained under arid and humid climatic conditions [5]. In particular, the data of the experimental station of the Qatar Ministry of Municipal and Agricultural Affairs for the arid zone were approximated by the least squares method under S-plot 7 using the following empirical equation: *T*(*t*) = *T*_0_ + *a*·sin(2 × 3.14*t*/*b* + *c*), where *a* = 4.45, *b* = 23.85, *c* = 4.18, and *T*_0_ = 38.14. Numerical simulation in Matlab-7 software showed that the potential losses in this treatment make up 9–10% for 580 h (less than a month) and reach 33% of the initial content for three months regardless of the SSPH concentration (Figure 5A). The use of temperature data typical for the humid climatic conditions in the Moscow megapolis according to [4,26] with the spline approximation of the dependence *T*(*t*) for an analogous numerical simulation showed that the destruction of the hydrogel proceeds in this case more slowly than in the irrigated arid soils. About 10–13% of the initial SSPH content (rate) is lost during a vegetation season. However, this is also a relatively high rate in technological terms.

The results of modeling the SSPHs biodegradation obtained above are in good agreement with the experimental estimate for SSPHs in urban soil constructions (Figure 5B). For a 0.1% dose of SSPH *№*2 at the soil surface, an experimental estimation in Moscow’s humid conditions yields a biodegradation constant *k* = 0.712 year^−1^ or a half-life *T*_0.5_ = 1 year. A numerical model of biodegradation [5], taking into account the dynamics of the temperature regime in a megacity, yields a biodegradation constant averaged over the vegetation season, *k* = 0.557 year^−1^ or a half-life *T*_0.5_ = 1.2 year, which is close to the experimental estimation. For arid conditions of irrigated agriculture with high temperatures of the summer vegetation period (40 ± 12 °C), the value of *T*_0.5_ decreases to 0.4 years, and the time for complete decomposition of the polymer *T*_0.95_ is 1.8 years. For the humid conditions of the Moscow megacity, the *T*_0.95_ estimate gives 4.2–5.2 years.

### 3.3. Dynamics of Technological Properties of Soil-Modifiers under the Influence of Biodegradation 

**Figure 6:**
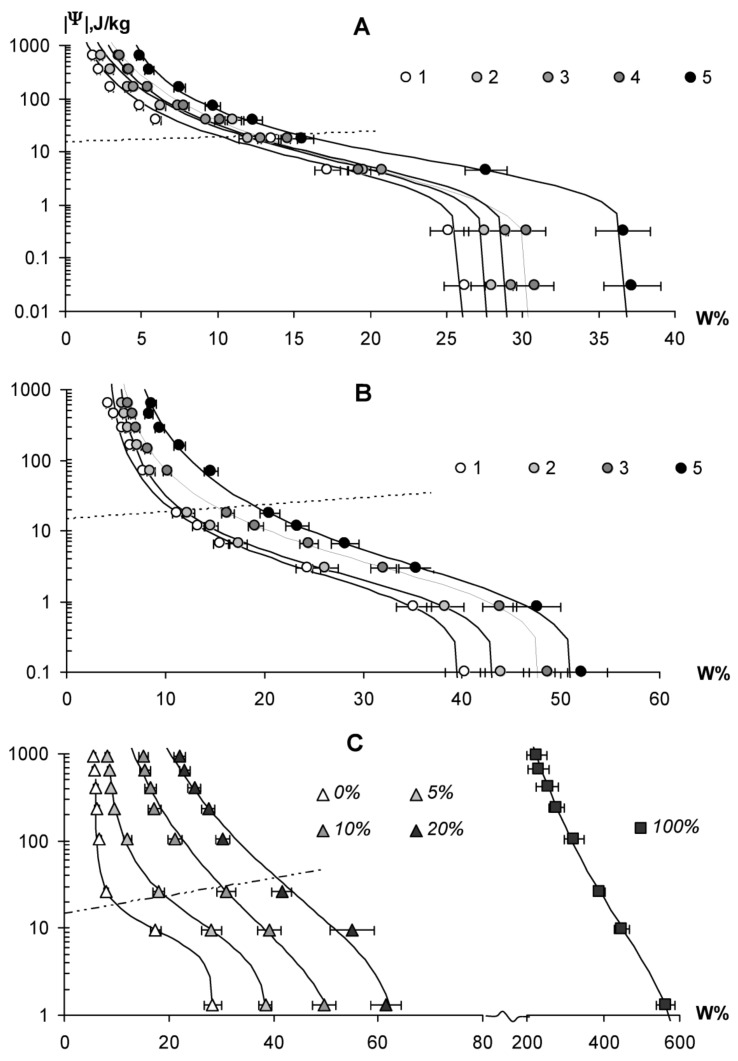
Dynamics of water-retaining capacity under the influence of soil conditioners and their biodegradation: (**A**) quartz sand, (**B**) silty-sand arenosol; 1–0% SSHP (control), 5–0.1% SSHP, experiment temperature: 2–37 °C, 3–30 °C, 4–20 °C; (**C**) peat soil modifier and its mixtures with monomineralic quartz sand; %—doses of the soil-modifier in the sand.

In advertising soil conditioners, their service life in soils is often indicated, in particular for hydrogels it is estimated at 4–5 years, that is, time close to *T*_0.95_. However, the linear biodegradation model (1), as well as real data, indicate rapid losses of biopolymers during the first year (Figure 2 and Figure 5). Hence, their technological qualities should deteriorate at the same rapid pace. Figure 6A,B shows the dynamics of water retention curves of a monomineral quartz substrate and a polymineral silty-sand arenosol under the action of a working dose of 0.1% of hydrogel *№*2 (radiation-crosslinked polyacrylamide) during the biodegradation experiment at temperatures of 20, 30 and 37 °C. The dotted line shows the moisture potential for determining the field moisture capacity, according to Voronin’s method [28]. Under the action of hydrogel, the physical state and water-retaining capacity of mineral coarsely dispersed substrates undergo strong changes. Hydrogels loosen the substrate, reducing bulk density of monomineral sand and arenesols from 1.3–1.5 g∙cm^−3^ to 1.1–1.3 g∙cm^−3^ and, respectively increasing the total moisture capacity (*W_s_*) by 10% or more from 26 to 40% in sand and from 37 to 52% in arenesols. The field water capacity increases by 6–10%, reaching values of 15% (sand) and 20% (arenesol), which is equivalent to a shift in the gradation by the granulometric composition from sand to sandy loams and light loams by water retention capacity.

However, all these technological results are almost completely lost within one year with biodegradation of the hydrogel at a temperature of 37 °C. The water retention curves and the *W_s_* = 28–44% as well as field moisture capacity (11–12%) calculated from them, in this case are statistically significantly different from the initial characteristics for the mineral substrates (Figure 6A,B). The smallest changes occurred at a minimum incubation temperature (20 °C). Here, the water retention curves and the calculated moisture capacity were reduced by approximately half the original state with 0.1% hydrogel concentration. The incubation variant at 30 °C occupied an intermediate position between the variants from 20 and 37 °C, and here the level of degradation of technological properties reached 2/3. All the results obtained were completely logical, starting from the well-known rule of Vant-Hoff, according to which the intensity of (bio) chemical processes increases by 2–3 times for every 10 °C of the temperature. The maximum temperature (37 °C) was, apparently, within the temperature optimum for a group of microorganisms that decompose the hydrogel; otherwise a decrease in the intensity of the biodegradation was evident.

A similar conclusion about the significant loss of technological properties of soil conditioners in biodegradation follows from the comparison of data on the rate of decomposition of peat soil-modifiers (Figure 2) and their water-retaining capacity in mixtures with mineral sand substrates (Figure 6C). Losses from biodegradation in 10% of organic matter are equal to a minimum twofold decrease in the water-holding capacity of peat-sand mixtures containing 10–20% of peat. Quite likely in a year, 50% of the loss from biodegradation for a pure 100% soil-modifier also corresponds to 2–3 fold losses of moisture capacity and water retention energy, that is, significant technological deterioration.

### 3.4. Inhibition of Hydrogels Biodegradation by Silver Remedies

Introduction of silver inhibitors of the biological activity significantly reduces respiration and biodegradation in pure gel-silver compositions (Figure 3 and Figure 4). In the case of silver ions under the action of a dose of 10 ppm *U_m_* decreases at 13–30 times. The exception is the gel Aquasorb where *U_m_* is reduced to not more than three times. Doses of 100–1000 ppm inhibit *U_m_* 20–60 times without significant differences on the effects between them. As the result calculated values for the effective half-lives *T*_0.5_ increase to 5–30 years in SSPH compositions with 10 ppm Ag and up to 25–50 years when the content of silver ions ranges from 100 to 1000 ppm. Silver nanoparticles have the same or a somewhat greater inhibitory effect. At the dose of 10 ppm Ag the value *U_m_* is equal 6–25 mg∙kg^−1^∙h^−1^ and *T*_0.5_ varies from 6 to 20 years, and at doses of 100–1000 ppm *U_m_* is equal 1.8–2.8 mg∙kg^−1^∙h^−1^ and *T*_0.5_ = 24–105 years.

In the case of mixtures of hydrogels with calcined sandy substrate and extracts from root rot, the silver inhibitors also strongly alter the biological activity and biodegradation rate of SSHP. Incorporation of ionic silver is effective only with large doses of 100–1000 ppm, which reduce *U_m_* in 5–10 times to values of 0.3–1.0 mg∙kg^−1^∙h^−1^ (Figure 4A). The value *T*_0.5_ increases in this case up to 1.7–5 years. A low dose of silver ions at 10 ppm, on the contrary, stimulates biological activity, which is probably due to the adsorption of Ag + on the negatively charged surface of soil particles and to the growth of microflora under the influence of nitrate anions remaining in the soil solution. Silver nanoparticles have a more powerful inhibitory effect in mixtures of SSPH with soil substrate and root rot compared to the ionic forms (Figure 4B). Under the influence of a low dose of 10 ppm Ag the index of biological activity *U_m_* is reduced to 6–40 times to values of 0.4–2.6 mg∙kg^−1^∙h^−1^. However, the calculated values of *T*_0.5_ in this case remained low and did not exceed 1.1 ± 0.7 years, except for sample *№*5 with peat filler. For this sample *U_m_* = 0.38 ± 0.04 mg∙kg^−1^∙h^−1^ and *T*_0.5_ = 3.7 ± 0.2 years. Higher doses of colloidal silver 100 ppm suppress *U_m_* to values of 0.03–0.30 mg∙kg^−1^∙h^−1^ except for sample *№*2 where the *U_m_* = 1.7 ± 1.2 mg∙kg^−1^∙h^−1^. Such values of biological activity corresponded to half-lives of hydrogels *T*_05_ from 5 to 50 years. A maximum dose 1000 ppm leads to *U_m_* about 0.01 ± 0.03 mg∙kg^−1^∙h^−1^, i.e., biological activity here is not statistically significantly different from zero. Calculated value T_0.5_ in this case varied from 52 ± 10 to 105 ± 60 years. In general, despite the strong variation in the obtained estimates of the intensity of SSHP biodegradation, the results of incubation experiments (Figure 3 and Figure 4) clearly confirm the increase in resistance of all types of hydrogels under the influence of silver inhibitors of biological activity. Presumably, the most effective doses of ions and silver nanoparticles are in the range of 10–100 ppm.

### 3.5. Dependence of Biodegradation Rate on Soil Depth and Nomographs for Assessing the Stability of Biopolymers in Urban Soil Design 

**Figure 7:**
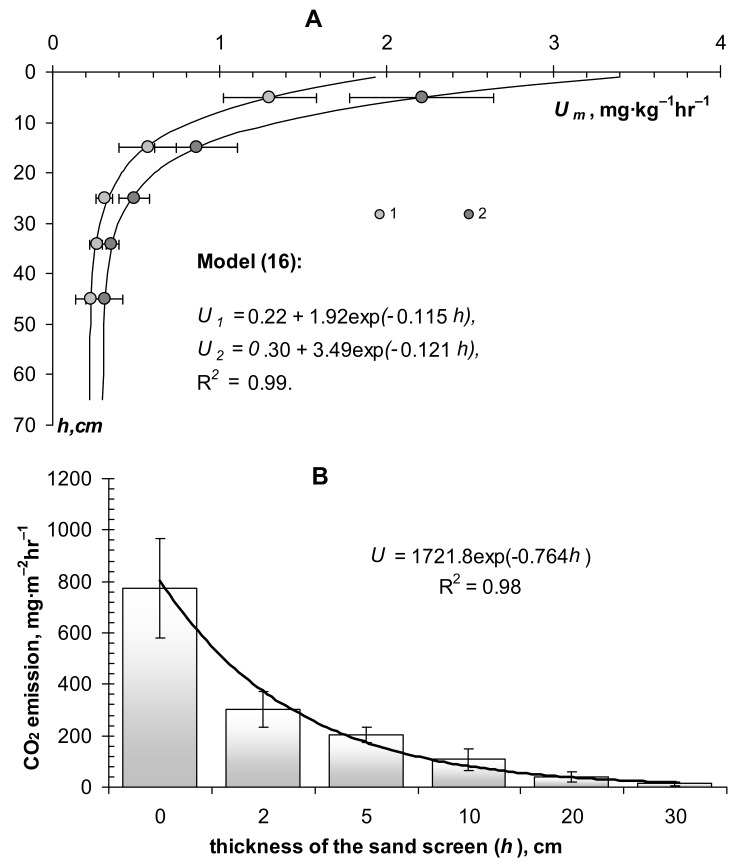
Dependence of biological activity (respiration) on the depth of the soil (**A**) and on the thickness of the sand layer covering the soil surface (**B**).

In previous works, we investigated the dependence of biological activity (respiration) on soil depth (*h*, cm) [2,4]. This dependence often obeys an exponential model of the form:

*U_m_*(*h*) = *U*_0_ + *a*·exp(−*bh*), (13)

where *U*_0_, mg∙kg^−1^∙h^−1^, *a*, mg∙kg^−1^∙h^−1^; *b*, cm^−1^ are empirical constants (Figure 7). The upper figure represents the results of the assessment of the biological activity for different layers of sod-podzolic loamy soil at the experimental station of the Moscow State University “Chashnikovo” using the usual method of incubation (*U*_1_) and its modification with thermal desorption and degassing of carbon dioxide (*U*_2_). Figure 7B shows the results of respiration assessment by standard closed-chambers method in same soil, covered by sand’s layer (sandy screen on surface of the soil) depending on its thickness. In both cases, for natural soil and artificial construction on its basis, biological activity decreases exponentially with depth, according to the model (13). Similar models are known for the distribution of roots and microorganisms in soils [22]. Apparently, the exponential function (13) with a rapid decline in biological activity with distance from the soil surface reflects the specific distribution of living organisms, organic substrates, as well as oxygen diffusing out of the atmosphere, that is, all the necessary components of aerobic biodegradation. The parameter *b*, which is responsible for the intensity of the decline in biological activity with depth for most mineral soils, excluding chernozems, varies in the range 0.1 ≤ *b* ≤ 1 cm^−1^. By analogy with the characteristic times of biodegradation (2), (3), for (13) it is possible to introduce indicators of characteristic depths of 50%, and 95% concentration of biological and biochemical sources of aerobic destruction of organic substances in soil [30] *H*_0.5_ = ln(2)/*b*, *H*_0.95_ = 3/*b*. For the above range *b*, they will vary from 1 to 7 cm (*H*_0.5_) (and from 3 to 30 cm (*H*_0.95_). A sharp (exponential) decrease in the intensity of biological activity with depth means the same decrease in the rate of biodegradation of organic substances when they are removed from the soil surface. Formula (14) allows, in a first approximation, to estimate at what depth (*H*) it is necessary to arrange the soil modifier in order to reduce its biodegradation by a predetermined number of times (*n*) with respect to decomposition on the soil surface [4]:

(14) $$H=\frac{\ln(n)}{b}$$

**Figure 8:**
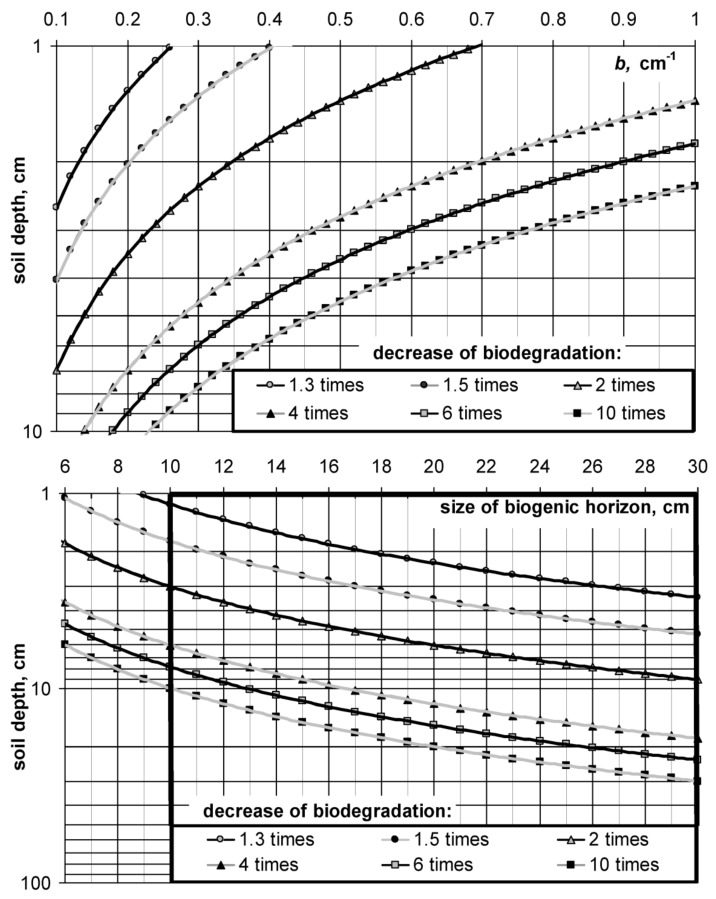
Nomographs for assessing the effect of deepening of organic soil-modifiers on their biodegradation in soils and soil constructions, according [4].

For the convenience of calculations, we propose using nomographs that allow us to estimate how many times the decomposition of the biopolymer material will decelerate when it is buried at some distance from the soil surface (Figure 8). The upper nomograph uses the exponential rate of decrease in biological activity with depth (*b*), according to the model (14). The known value of *b*, plotted on the abscissa axis, gives a point on the graphic line, the ordinate of which represents the required depth. For example, if *b* = 0.35 cm^−1^ and we want to reduce biodegradation by 4 times (the fourth line from the top), then the depth *H* will be 4 cm. 

Since the exponent *b* must be determined experimentally, in the simplified version of the nomograph (lower Figure 8), it is possible to make an estimate by the size of the zone of the most active microbial transformation of organic substances (*H*_0.95_). There is a reason to identify this value with the size of the biogenic (humus) soil layer, which for most soils, excluding chernozems and peat bogs, usually does not exceed 20–30 cm [30]. Therefore, in the nomograph, the range of variation in the size of the biogenic soil horizon is determined by the inequality 6 ≤ *H*_0.95_ ≤ 30 cm. The calculation by simplified nomograph is similar to the previous one. For example, if in the urban soil the biogenic, humified horizon had a thickness of 20 cm, then biopolymer material must be placed at a depth of *H* = 6 cm in order to reduce the biodegradation intensity twice (third line of the nomograph from the top).

Obtained results of the technological modeling were successfully confirmed by practical experiments carried out on layered soil constructions for lawns in the conditions of Moscow megacity (Figure 5B,C). Shielding of soil-modifiers by a 5 cm layer of sand or the so-called “sanding of the lawn” led to the conservation of organic matter and its protection from biodegradation. The decrease in the kinetic constants of destruction (*k*) was 4–5 times (from 0.71 year^−1^ to 0.17 year^−1^ in the case of SSHP and from 0.54 year^−1^ to 0.12 year^−1^ for peat). The corresponding losses of organic matter per year compared with the decomposition rate on the surface decreased by 3.8 times for a peat soil modifier, and by 3.2 times for a SSHP. This practical result is in good agreement with the theoretical calculation of the proposed nomogram method. With the size of the biologically active layer with organic conditioners (*H*_0.95_) in soil constructions of 10 cm, according to the lower nomograph (Figure 8), the intensity of destruction in the case of 5–6 cm of the screen should decrease by 3–4 times, which was actually observed.

## 4. Discussion

### 4.1. Biodegradation of Peat

Analysis of the known information on the rate of biodegradation of peat and peat-mineral mixtures gives contradictory data. The estimates given in the “Introduction” of *T*_0.5_ = 5–50 years and *T*_0.95_ up to 250 years for peat of the upper (active) layer in boreal and tropical bogs refer to the natural moistening conditions. Perhaps these values even overestimate the intensity of this process. Latter at al. [31], studying the decomposition of the predominant plant litters on Pennine moorland in northern England during long-term 23 years experiment, found that the actual rate of biodegradation is lower compared to estimates by the incubation method and in short-term field experiments. A similar conclusion about the strong deceleration of biodegradation for a long time was obtained in the work on mathematical modeling of this process [32]. However, in all the cases analyzed above, biodegradation of peat was restrained by anaerobic conditions in natural bog ecosystems or in artificial incubation experiments. Drainage (aeration) greatly accelerates biodegradation and under aerobic conditions, peat loses its resistance to decomposition. According to Figure 1, only a small decrease in peat moisture to 0.6–0.7 *W_s_* is sufficient for a sharp increase in destruction. As a result, drained peatlands degrade rapidly and lose catastrophically the main amount of organic matter in the first 5–10 years at a rate of up to 10–12 t·ha^−1^·year^−1^ [16] or more than 2% *C*·year^−1^ in soils with carbon contents greater than 100 g·kg^−1^ [1]. Such lands, because of the negative carbon balance, are converted into intensive sources of carbon dioxide [33]. By analogy, the high rate of CO_2_ production by urban soils may be associated with an increased content of organic substrates, including peat-sand mixtures for gardening [4,26,34,35]. The high contribution of soil cover to urban CO_2_ emissions is shown experimentally in [36]. According to this study, summer CO_2_ emissions from soils in the residential areas of Greater Boston (USA) can reach 72% of the anthropogenic source (fuel combustion).

Reverse positive relationship of biodegradation with temperature increases peat decomposition under local greenhouse effect conditions [37,38]. For cities such a factor may be the so-called “urban heat island” [26,39]. In this case, the period of biological activity is prolonged for the cold season and biodegradation of peat during winter thaws can reach 20–25% of average annual losses (see “Results”). Additional stimulation of biodegradation of peat and peat mixtures in the megalopolis is also possible due to the priming effect [18], for example, from easily degradable plant residues in the form of mown lawn grass, faeces of domestic animals and organic debris. All these facts explain the obtained above high values of biodegradation rate of peat soil-modifiers in urban environments, with loss of organic matter, up to 50–60% in the first year, with the half-lives *T*_0.5_ about 1 year and *T*_0.95_ not more than 4 years.

### 4.2. Biodegradation of Synthetic Hydrogels and Their Components

On the problem of biodegradation of SSHP, published sources give meager and very contradictory data concerning mainly acrylamide (AAM) and polyacrylamide (PAA). We exclude from consideration numerous advertising information about the stability of SSHP in soils for 5–6 years or more, because in our opinion they are not correspond to reality. Lentz et al. [40] reports a rather slow biodegradation of PAA not more than 10% year^−1^ primarily through the shear-induced chain scission and photodegradation. According to the exponential model (1–3), this corresponds to k = 0.11 year^−1^. In this case, the half-life of PAA hydrogels *T*_0.5_ = 6.6 years and *T*_0.95_ = 28.5 years, which significantly exceeds our results *T*_0.5_ = 1–1.2 years and *T*_0.95_ = 4.2–5.2 years for the radiation-crosslinked PAA (sample *№*2) obtained in this work and previous publication [5]. 

However, there are other data that offer an alternative perspective. Some studies found that polyacrylamide (PAM) and acrylamide monomer degradation in soil is very rapid with half-life values about few days [41,42,43,44,45]. Sojka and Entry [45] have reported that PAM was completely degraded within five days after applying 500 mg kg^−1^ garden soil. Lande et al. [41] have estimated the half-life of acrylamide monomer in agricultural soils ranged from 18 to 100 h at a concentration of 25–500 mg kg^−1^ and a temperature of 20–22 °C. Increasing the initial concentration or decreasing the temperature have increased the half-life [41,42]. The use of PAA and other acrylic hydrogels had no adverse effects on soil microbial communities and often statistically significantly increased the respiration of the soil (CO_2_ emission) [4,46,47]. Soil microorganisms are capable of utilizing PAM or acrylamide as a source of nitrogen [42,44,45]. As shown in [42] acrylamide monomer is subject to biodegradation in soils and its transformation is assumed to be due to amidase activity. All these facts indicate a low potential stability of acrylic hydrogels in soils and the dominant mechanism of their biological (biochemical) degradation, rather than chemical or photochemical decomposition. The main controlling factors of biodegradation of acrylic SSHP are their composition, temperature and the depth of localization in the soil [4,5]. Soil moisture is not so important, because hydrogels almost always contain water available for microorganisms. While the large changes in soil humidity that cause the transition from anaerobic to aerobic conditions, for example in rice paddies, strongly influence the biodegradation of SSHP, increasing it after aeration of the soil [40].

Data on the effect of microflora inhibitors on SSHP biodegradation have not been found and, apparently, our studies are pioneering in this area. However, the values of the effective concentrations of ions and silver nanoparticles in 10–100 ppm obtained in our research are in good agreement with the published results [48,49,50] for fungicide and bactericide efficiency of aqueous and colloidal silver solutions with an effective concentration range of 1–50 up to 100 ppm. In the soil, for which protective hydrogel compositions are designed, effective concentration of silver suppressing the growth of plants vary in the range of 50–1000 mg/kg of the solid phase [51]. In terms of soil solution it gives at the range from 250 to 5000 ppm (20% water content). Shclich et al. [52] reported similar values from 200 to 400 ppm for earthworms in the soil at experimental doses of ionic and colloidal silver from 15 to 1000 mg/kg or from 75 to 5000 ppm at 20% soil moisture. Even if we assume an unlikely event of leaching of all silver with fast biodegradation of SSHP, the concentrations in the soil solution of 10–100 ppm will not exceed the threshold, which is dangerous for plants and soil invertebrates. 

### 4.3. Dependence of Biodegradation Intensity on Soil Depth

Despite the obvious idea of reducing the rate of biodegradation of organic substances with soil depth, quantitative studies in this area are practically absent. Strong differences in the characteristic times of biodegradation of organic soil components are usually explained only by their composition and association with mineral components. There is general agreement in soil science that the distribution of organic matter decomposition rates tends to cluster at three very different time scales: Subannual, decadal-century, and longer [3,14]. This idea is supported by almost all modern models of dynamics of soil organic matter [32]. Fresh litter compounds, microbial cell, root exudates or so-called “active pool” decompose on time scales of hours or months to years. Highly stabilized organic matter, typically associated with mineral surfaces or very stable aggregates, persists in soils for thousands of years and is often referred to as the “passive” or “millennial cycling C” pool. The remaining “intermediate” or “slow” pool has turnover times in the range of decades to centuries, and may consist of structural components of plant detritus more resistant to decay, or soil humus that have been stabilized by their association with soil minerals or aggregate structures [3]. We consider this approach to be incomplete and suggest supplementing it with information on the vertical differentiation of biodegradation of organic substances in soils. In the upper biological and chemically active soil horizon (no more than 10–20 cm for most soils), aerobic biodegradation is very strong and copes rapidly on a geological scale of time with any organic matter, including hard-decomposable (lignin, humus, bio-coal, asphaltenes, etc.). That is the reason why the average time of turnover of the organic matter of soils is estimated in tens of years, and the radiocarbon dating of the organic matter of the upper horizons does not exceed the first hundred years (see Introduction). Probably, rapid biodegradation of organic substances in the surface active layer of soil is associated with optimal thermodynamic conditions (*T*, *W*), maximum biodiversity and concentration of microorganisms, enzymes, free oxygen and substances that stimulate this process by the principle of priming effect [18]. Only at a distance from the soil surface can we find organic matter with the age of several thousand years by radiocarbon method, such as in chernozems, peat bogs or buried soils [3,11]. A sharp decrease in the rate of biodegradation with soil depth should be taken into account when designing soil constructions in order to increase the stability of organic substances and their service life [4,5]. For example, reclamation technologies for peat soils based on applying sand in doses of 200–1600 m^3^ ha^−1^ with subsequent mixing with peat give a significant increase in productivity but are often accompanied by acceleration of peat decomposition [16,53]. The layered method of sand introducing, or so-called “cover structure”, as it follows from our nomographs (Figure 8), reduces the intensity of biodegradation of peat material. According to the experimental data of Polesskaya experimental station (Belarusian research Institute of land reclamation and water management, Brest region, Belarus), peat coating with a layer of sand of 4 cm slows its decomposition by approximately 1.6 times, and with a layer size of 10 cm up to 2–2.5 times, and this effect is persistently maintained throughout the four-year field experience [16].

It is equally important, in our opinion, to introduce the idea of a sharp reduction in biodegradation with soil depth in the models for predicting the behavior of xenobiotics, in particular, pesticides in soils. Usually the penetration of pesticides into groundwater is mostly associated with preferential flows [54], whereas there is another mechanism, namely the conservation of pesticides at a distance from the active biodegradation layer with gradual subsequent leaching to the vadose zone. 

We would like to emphasize once again that absolutely stable organic substances for improving or impairing the quality of soil do not exist, and our ability to manage the ecological state of soils and create stable artificial soils is largely determined by the knowledge of the natural patterns of soil organic matter biodegradation. However, biodegradation of polymeric materials in soils is now extremely important in the utilization of numerous materials by mankind for their needs [55]. We hope that the approach proposed in the article to the quantitative assessment of biodegradation in soils will be useful for solving the problem of utilization of biodegradable polymers as environmentally safe materials/

## 5. Conclusions

1. Biodegradation of organic soil conditioners in biologically and chemically active soil environment is the main factor reducing their effectiveness and quality, most notably including water-retention capacity.
2. Traditionally used in urban landscaping, peat-based soil modifiers in Moscow conditions lose up to 50–60% of organic matter during the first year due to intensive biodegradation with a half-life *T*_0.5_ about 1 year and *T*_0.95_ not more than 4 years.
3. Incubation experiments with natural and synthetic polymers revealed an exponential dependence of the rate of biodegradation from temperature and more complicated dependence from humidity with a maximum near 0.6–0.7 *W_s_* (soil water saturation). For its description, a new two-parameter empirical model is proposed.
4. Modern soil conditioners in the form of synthetic polymeric hydrogels based on polyacrylamide and acrylates are susceptible to microbial degradation and are characterized by half-lives of 0.5 to 2.6 years (to 5.2 ± 1.5 years in the case of the SSPH filling by dispersed peat).
5. Mixing hydrogel compositions with a mineral soil substrate and putrefactive micro-organisms leads to a sharp increase of biological activity and biodegradation of SSPH; their half-lives not exceed one year.
6. The protective composition on the basis of hydrogels and silver inhibitors are more resistant to biodegradation. Calculated half-lives in them with a small dose of silver in 10 ppm range from five to 30 years, and with large doses of 100–1000 ppm can reach 25–50 years and above. Silver nanoparticles have generally stronger inhibitory effect compared to ions.
7. The rapid (exponential) decrease in the intensity of biodegradation with the depth of the soil makes it possible significantly (2–4 times or more) increase the stability of organic soil-modifiers if they are located at a distance from the soil surface in the process of creating a layered urban constructozems.

## 6. Patents

The results of the work are used in the synthesis technology of biodegradation-resistant filled hydrogels patented in the Russian Federation:

Patent RU 2536509 (http://www.findpatent.ru/patent/253/2536509.html, 21 June 2018);

Patent RU 2639789(http://www.findpatent.ru/patent/263/2639789.html, 21 June 2018).
